# Comparison of suicide risk across medical specialties, and the examination of the prevalence of depressive symptoms and hopelessness among Hungarian physicians

**DOI:** 10.1192/j.eurpsy.2026.10684

**Published:** 2026-07-31

**Authors:** K. Kostyál, O. Csabai, T. T. Simonyi, I. K. Pribék, K. M. Hegedűs, D. Varga, A. Kukla, B. Paksi, Z. Demetrovics, P. Z. Álmos

**Affiliations:** 1 Department of Psychiatry, Albert Szent-Györgyi Medical School, University of Szeged, Szeged; 2 Institute of Education, ELTE Eötvös Loránd University, Budapest, Hungary; 3 Flinders University Institute for Mental Health and Wellbeing, College of Education, Psychology and Social Work, Flinders University, Bedford Park SA, Australia; 4 Institute of Psychology, ELTE Eötvös Loránd University, Budapest, Hungary; 5 Centre of Excellence in Responsible Gaming, University of Gibraltar, Gibraltar, Gibraltar

## Abstract

**Introduction:**

More than 720,000 people die by suicide every year worldwide. Medical-related professions are at high risk. There is emerging evidence of a link between area of specialty and risk of suicide. Anaesthetists, psychiatrists, general practitioners and general surgeons might have higher rates of suicide than other specialists. Mental health issues, especially hopelessness and depressive symptoms have been shown to increase the risk of suicide among doctors as well.

**Objectives:**

The objective of this research was to evaluate the severity of depressive symptoms and hopelessness among physicians, and to examine how medical specialties affect suicide risk in our sample.

**Methods:**

The study consisted of two data collection phases, with 8,000 participants randomly selected from the members of the Hungarian Medical Chamber. In addition to doctors, chamber members may include dentists, licensed specialist psychologist, and medical scientists. In this abstract, the results from the Beck Hopelessness Scale, Patient Health Questionnaire-9, and Columbia Suicide Severity Rating Scale (Screen Version) and demographic items were used. The data analysis included descriptive statistics, frequency analyses and ordinal logistic regression.

**Results:**

In this study, the responses of 1,380 participants were analyzed. The sample had a mean age of 51.32 years (SD = 15.36), and 68.2% were female. Among the respondents, 82.3% reported mild, 14.3% moderate, and 3.5% severe hopelessness symptoms. 25.7% of respondents reported experiencing symptoms at least at the level of moderate depression. According to suicide risk, respondents were classified into the following categories: 82.9% no risk, 7.6% low risk, 7.4% moderate risk and 2.2% high risk.

The ordinal logistic regression showed adequate fit, but overall did not significantly improve prediction compared to the null model (likelihood ratio test: χ²(9) = 15.49, *p* = 0.078). Pseudo R² values (Cox & Snell = 0.014; Nagelkerke = 0.019; McFadden = 0.011) indicated that medical field explained only a small fraction of variance in suicide risk. Parameter estimates showed that most professional fields did not differ significantly from the reference category (dentistry). Two fields showed significantly lower risk compared to dentistry, namely general practice (OR = 0.57, p = 0.042) and psychology (OR = 0.09, p = 0.019).

**Conclusions:**

This research highlights the prevalence of hopelessness and depression among Hungarian physicians, and suggest that medical specialties have limited predictive value for suicide risk levels. The majority of physicians experience mild hopelessness, a notable proportion face moderate to severe levels. Depressive symptoms probably affect daily life and well-being in a quarter of doctors. These findings underline that physicians are not immune to mental health challenges and need targeted interventions.

**Disclosure of Interest:**

K. Kostyál Grant / Research support from: Hetényi Géza grant (5S764 A202), University Szeged SZAOK Faculty Research Found., O. Csabai Grant / Research support from: Hetényi Géza grant (5S764 A202), University Szeged SZAOK Faculty Research Found., T. Simonyi Grant / Research support from: Hetényi Géza grant (5S764 A202), University Szeged SZAOK Faculty Research Found., I. Pribék Grant / Research support from: Hetényi Géza grant (5S764 A202), University Szeged SZAOK Faculty Research Found., K. Hegedűs Grant / Research support from: Hetényi Géza grant (5S764 A202), University Szeged SZAOK Faculty Research Found., D. Varga Grant / Research support from: Hetényi Géza grant (5S764 A202), University Szeged SZAOK Faculty Research Found., A. Kukla Grant / Research support from: Hetényi Géza grant (5S764 A202), University Szeged SZAOK Faculty Research Found., B. Paksi Grant / Research support from: Hetényi Géza grant (5S764 A202), University Szeged SZAOK Faculty Research Found., Z. Demetrovics Grant / Research support from: Hetényi Géza grant (5S764 A202), University Szeged SZAOK Faculty Research Found., P. Álmos Grant / Research support from: Hetényi Géza grant (5S764 A202), University Szeged SZAOK Faculty Research Found.

